# Discordance Between Spatial and Population Correlations From Human Brain Imaging Data

**DOI:** 10.1002/hbm.70421

**Published:** 2025-11-26

**Authors:** Patrick M. Fisher, Kristian Larsen, Pontus Plavén‐Sigray, Gitte M. Knudsen, Brice Ozenne

**Affiliations:** ^1^ Neurobiology Research Unit Copenhagen University Hospital Rigshospitalet Copenhagen Denmark; ^2^ Department of Drug Design and Pharmacology University of Copenhagen Copenhagen Denmark; ^3^ Department of Clinical Medicine University of Copenhagen Copenhagen Denmark; ^4^ Department of Clinical Neuroscience, Centre for Psychiatry Research Karolinska Institutet and Stockholm Health Care Services, Region Stockholm Stockholm Sweden; ^5^ Section on Biostatistics, Department of Public Health University of Copenhagen Copenhagen Denmark

**Keywords:** correlation, human brain imaging, magnetic resonance imaging, positron emission tomography, regression, statistical analysis

## Abstract

It has become increasingly common to probe correlations between human brain imaging measures of receptor/protein binding and function using population‐level brain maps, typically drawn from independent cohorts to estimate correlations across regions. This strategy raises issues of interpretation that we highlight here with both an empirical multimodal brain imaging dataset and simulation studies. Twenty‐four healthy participants completed neuroimaging with both [11C]Cimbi‐36 positron emission tomography and magnetic resonance imaging scans to estimate receptor binding potential (BP) and cerebral blood flow (CBF), respectively, in 18 cortical/subcortical regions. Correlations between BP and CBF were estimated in four ways: (1) Pearson correlation across regions of mean regional BP and CBF from a single or separate cohorts ($\rho_{1.1}$ and $\rho_{1.2}$, respectively), to mimic studies using data from independent cohorts; (2) Pearson correlation between BP and CBF across participants in each region ($\rho_{2}$); or (3) the correlation between BP and CBF across participants across all regions within a single linear mixed effects model ($\rho_{3}$). We observed a significant positive correlation across regions (${\hat{\rho }}_{1.1}$ = 0.672, *p* = 0.0023; ${\hat{\rho }}_{1.2}$ = 0.659, *p* = 0.0030). Region‐specific correlations across participants were substantively lower and not statistically significant (${\hat{\rho }}_{2}$: mean = 0.140, range = −0.112–0.336; all *p* > 0.10), nor when estimated simultaneously within a linear mixed model (${\hat{\rho }}_{3}$ = 0.138, *p* = 0.26). Our simulation study illustrated that regional differences in BP or CBF mean and variance can substantially bias across‐regions correlations and inflate the type‐1 error rate. Our observations allude to ambiguity in the meaning of across‐regions correlations and suggest interpreting them as evidence for a biological relation, which implies a relation across participants, is problematic. Without validated methods that handle confounding and other biases, we urge caution in how future studies interpret across‐regions correlations of population‐level brain maps.

## Key Points

- Correlation analyses across regions made between population‐level brain imaging maps are common yet raise issues of interpretation.
- We show that such correlations do not align with across‐participants correlations, evidencing high bias and inflated type‐1 error rates.
- Across‐regions correlations have an ambiguous meaning and are not straight‐forward proxies for across‐participants correlations.

## Graphical abstract

We show that the across‐regions correlation of population‐level brain maps does not align with the typically desired across‐participants correlation. Across‐region correlations are variably biased with potentially large type‐1 error rates, raising issues of their interpretation and generalizability.

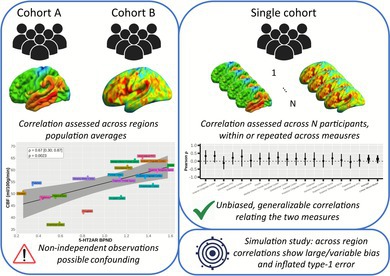

## 1 Introduction

Functional brain imaging tools, e.g., blood oxygen level dependent functional magnetic resonance imaging (BOLD fMRI), arterial spin labeling (ASL) MRI, electroencephalography, magnetoencephalography, and functional near‐infrared spectroscopy, can be applied to assess individual task‐related and task‐independent activation and connectivity patterns across the brain under baseline conditions or following an intervention within healthy or clinical cohorts. Despite the wealth of brain‐related information that can be gained from these tools, they are not without limitations. One such limitation is that it is not possible to directly measure molecular mechanisms (e.g., specific neurotransmitters or receptors) associated with an observed activation pattern or correlation structure. This information can be highly relevant for many reasons, e.g., knowledge of critical receptor pathways can guide a pharmacological intervention study targeting that receptor. Molecular brain imaging tools, e.g., positron emission tomography (PET) and single‐photon emission computed tomography (SPECT) can illuminate the spatial distribution of specific neuromolecular processes, e.g., glucose metabolism, receptor binding or enzymatic activity. As such, acquiring both functional and molecular brain imaging data within a single cohort can implicate specific molecular mechanisms in shaping relevant brain function (Fisher and Hariri 2013; Fisher et al. 2006; Sankar et al. 2023). Unfortunately, such a multimodal design is not feasible in many research settings. Molecular brain imaging is especially resource demanding, requiring a specialized infrastructure, i.e., cyclotron and radiochemistry laboratory, with costs that can be prohibitively expensive. Subsequently, researchers have sought to develop alternative strategies for mapping neuromolecular mechanisms onto brain activity and connectivity patterns.

The recent push to make more biomedical research data publicly available has contributed to the emergence of valuable resources such as the Allen Brain Atlas and other online repositories wherein one can obtain organ or brain‐region specific levels of gene expression and/or protein concentrations. Similarly, human molecular imaging research groups are making publicly available more and more population‐level brain‐wide maps depicting, e.g., receptor or transporter levels (Beliveau et al. 2017; Johansen et al. 2024; Nørgaard et al. 2021; Savli et al. 2012). Subsequently, there has been a rapid expansion in studies leveraging these publicly available brain atlases, typically in conjunction with functional brain maps, to establish evidence of molecular mechanisms underlying aspects of brain function (see Table S1 for a non‐exhaustive list of > 40 such studies). In almost all cases, these association studies cannot be performed at the participant level because the two brain maps are derived from independent cohorts. Instead, a typical strategy is to average the functional and molecular brain maps across the respective participants for a common set of regions. These association studies go beyond the typical use of atlases as a descriptive tool. A statistical analysis is then made *across regions* where a statistically significant correlation is inferred as evidence that the relevant molecular feature has some biological relation to the functional brain signal, implying a relation that generalizes to broader populations, e.g., healthy individuals or specific diagnostic groups. Likewise, vice versa, the absence of evidence for an association between the two measures implicitly suggests a limited or negligible relation between the molecular and functional constructs.

Concerns about an inflated type‐1 error rate of this method have been noted previously and strategies aimed at addressing this limitation have been described, e.g., spatial permutation framework (Alexander‐Bloch et al. 2018). However, there are reasons to believe that this interpretation of across‐regions correlations is more fundamentally problematic and that such analyses do not inform about relations that are generalizable across individuals, irrespective of statistical significance correction strategies. Essentially, a spatial correlation between signals can exist in the absence of a biological relation between those signals. Here we highlight these pitfalls through a multimodal human brain imaging dataset including serotonin 2A receptor (5‐HT2AR) binding with [11C]Cimbi‐36 PET and cerebral blood flow with ASL MRI as well as an associated simulation study. We also elaborate on the statistical principles underlying why this inference is problematic.

## 2 Materials and Methods

### 2.1 Participants

Here we report data from 24 healthy volunteers (10 females; age, mean ± sd [range] = 32.5 ± 7.9 [24–58] years) who participated in a broader study related to neural effects of serotonin 2A receptor (5‐HT2AR) signaling (NCT03289949). Participants were generally healthy and without current or previous personal or family history of psychiatric or neurological disease. Detailed inclusion/exclusion criteria have been reported previously (Madsen et al. 2021). Measures and related information were extracted from the Cimbi database following an approved database application (https://nru.dk/index.php/allcategories/category/224‐cimbi) (Knudsen et al. 2016). Prior to inclusion, participants received written and verbal descriptions of the study protocol; all participants provided written consent prior to study participation. The overall study was approved by the ethics committee for the Capital Region of Copenhagen (journal identifier: H‐16028698, amendments: 56023, 56967, 57974, 59673, 60437, 62255) and Danish Medicines Agency (EudraCT identifier: 2016‐004000‐61, amendments: 2017014166, 2017082837, 2018023295). Cerebral blood flow and 5‐HT2AR non‐displaceable binding potential (BPND) estimates used in this article have been reported in previous, unrelated publications (Larsen et al. 2025; Søndergaard et al. 2022; Spies et al. 2020).

### 2.2 Magnetic Resonance Imaging

The acquisition and preprocessing methods are described below and the same as described previously (Larsen et al. 2025). In brief, participants were scanned on one of two 3 T Siemens Magnetom Prisma MRI scanners (Siemens AG, Erlangen, Germany) at Rigshospitalet, Copenhagen (MR1 and MR2). Data were drawn from two scanners because MR1 became unavailable during data collection. Fifteen participants completed scan sessions on MR1 and nine participants on MR2. All images were visually inspected for quality using the FSL image viewer, FSLeyes (Jenkinson et al. 2012).

#### 2.2.1 MR1 Acquisition Parameters

Imaging data were acquired using a 64‐channel head/neck coil. We acquired a high‐resolution, T1‐weighted 3D MPRAGE structural image (inversion time = 900 ms, echo time = 2.58 ms, repetition time = 1900 ms, flip angle = 9°, in‐plane matrix = 256 × 256, in‐plane resolution = 0.9 mm × 0.9 mm, number of slices = 224, slice thickness = 0.9 mm, no gap). We acquired a pseudo‐continuous arterial spin labeling (pcASL) sequence to estimate CBF using a 2D echo‐planar imaging (EPI) gradient spin‐echo sequence (scan time = 332 s, echo time = 12 ms, repetition time = 4000 ms, flip angle = 90°, label duration = 1508 ms, single post‐labeling delay = 1500 ms, in‐plane matrix = 64 × 64, in‐plane resolution = 3 mm × 3 mm, number of slices = 20, slice thickness = 5 mm no gap, number of control/label pairs = 40, slice readout duration = 35 ms). To calibrate the pcASL signal, an M0 scan was acquired using the same imaging parameters except for a repetition time of 10,000 ms. We acquired a gradient echo sequence (GRE) to estimate an underlying field map to correct for geometric distortions in the pcASL images (echo time 1 = 4.92 ms, echo time 2 = 7.38 ms, repetition time = 400 ms).

#### 2.2.2 MR2 Acquisition Parameters

Imaging data were acquired using a 32‐channel head coil. We acquired a high‐resolution, T1‐weighted 3D MPRAGE structural image (inversion time = 920 ms, echo time = 2.41 ms, repetition time = 1810 ms, flip angle = 9°, in‐plane matrix = 288 × 288, in‐plane resolution = 0.8 × 0.8 mm, slice thickness = 0.8 mm, number of slices = 224, no gap). We acquired pcASL with a five post‐label delay 3D turbo gradient spin echo sequence (scan time = 431 s, 12 label/control pairs, native voxel size: 2.5 × 2.5 × 3 mm, image matrix = 96 × 96 × 40, label duration = 1508 ms, post‐label delays = [500, 500, 1000, 1000, 1500, 1500, 2000, 2000, 2000, 2500, 2500, 2500] ms, echo time = 3.78 ms, repetition time = 4100 ms, flip angle: 120°, two background suppression pulses optimized for each PLD). The acquisition included an M0 scan with which to calibrate the pcASL signal without any background suppression. Spin‐echo echo‐planar acquisitions with opposite phase‐encoded blips were acquired to correct for geometric distortions using *topup* implemented in FSL (FMRIB software library, www.fmrib.ox.ac.uk) (Andersson et al. 2003; Smith et al. 2004).

### 2.3 Quantification of Cerebral Blood Flow

#### 2.3.1 MR1 Processing

The pcASL data underwent intramodal registration, dividing label and control images (40 pairs) before realigning them. The pcASL data were then unwarped to correct for B0 field distortions. Realignment and distortion correction were performed using SPM12 (https://www.fil.ion.ucl.ac.uk/spm/software/spm12/). The high‐resolution T1‐weighted structural image was coregistered with the realigned and motion corrected pcASL data. We corrected for the interleaved slice acquisitions in the 2D EPI protocol using a delay in slice time acquisition of 35 ms.

#### 2.3.2 MR2 Preprocessing

For each PLD, a label/control difference image was realigned and the high‐resolution T1‐weighted structural image was co‐registered with the M0 image. Co‐registration with the pcASL images as we did for the MR1 data was not possible without introducing transformation artefacts.

All pcASL images from both scanners were quantified to absolute CBF values using BASIL in FSL (FMRIB Software Library, www.fmrib.ox.ac.uk), which estimates absolute CBF (mL/100 g/min) using a single, well‐mixed tissue compartment model (Buxton et al. 1998; Chappell et al. 2009). We used standard settings for the quantitative modeling. Following segmentation of the high‐resolution T1‐weighted structural image in SPM12 to produce gray‐matter, white‐matter, and cerebrospinal fluid probability maps, mean gray‐matter‐probability‐weighted regional CBF estimates were computed (see below for region definition).

### 2.4 Positron Emission Tomography

[11C]Cimbi‐36 was synthesized as previously reported (Ettrup et al. 2014). All PET scans were acquired using a Siemens ECAT High Resolution Research Tomograph (HRRT, CTI/Siemens, Knoxville, TN) in 3D acquisition mode and with an in‐plane resolution of < 2 mm. After securing the participant's head in a head holder with cushions to limit motion, scans were performed following a bolus radioligand administration, using an established protocol: a 6‐min transmission scan was followed by 120‐min (frames: 6 × 10s, 6 × 20s, 6 × 60s, 8 × 120 s, and 19 × 300 s) of dynamic PET scanning, which commenced with [11C]Cimbi‐36 injection (Ettrup et al. 2014, 2016). Scans were reconstructed using a 3D‐OSEM‐PSF algorithm (Hong et al. 2007; Sureau et al. 2008).

### 2.5 Positron Emission Tomography Processing

PET data were processed using Pvelab (Svarer et al. 2005). We applied the AIR algorithm to correct for motion during PET scans, wherein each PET scan was first smoothed with a 10 mm within‐frame Gaussian filter prior to the estimation of motion (Woods et al. 1992). Motion correction was applied to unfiltered PET frames. The high‐resolution T1‐weighted structural MRI scan was co‐registered into PET space and segmented into gray‐matter, white‐matter, and cerebrospinal fluid probability maps using SPM12 (https://www.fil.ion.ucl.ac.uk/spm/software/spm12/). Individual regions were defined in subject space using Pvelab and included: middle/inferior frontal gyrus, superior frontal gyrus, and superior temporal gyrus as well as occipital cortex, orbitofrontal cortex, parietal cortex, anterior cingulate cortex, insula cortex, posterior cingulate cortex, ventrolateral prefrontal cortex, dorsolateral prefrontal cortex, sensory‐motor cortex (i.e., pre/post‐central gyri), amygdala, hippocampus, entorhinal cortex, thalamus, putamen, and caudate. A mean time series was computed for each gray‐matter masked region to quantify [11C]Cimbi‐36 non‐displaceable binding potential (BPND) using the simplified reference tissue model (SRTM) with cerebellum as the reference region (Ettrup et al. 2014).

Subject‐specific CBF images were co‐registered to PET images using CBF‐aligned M0 images and PET‐aligned high‐resolution T1‐weighted structural images. Gray‐matter‐weighted regional CBF values were calculated within the 18 Pvelab‐defined, subject‐specific regions.

### 2.6 Brain Maps

**FIGURE 1:**
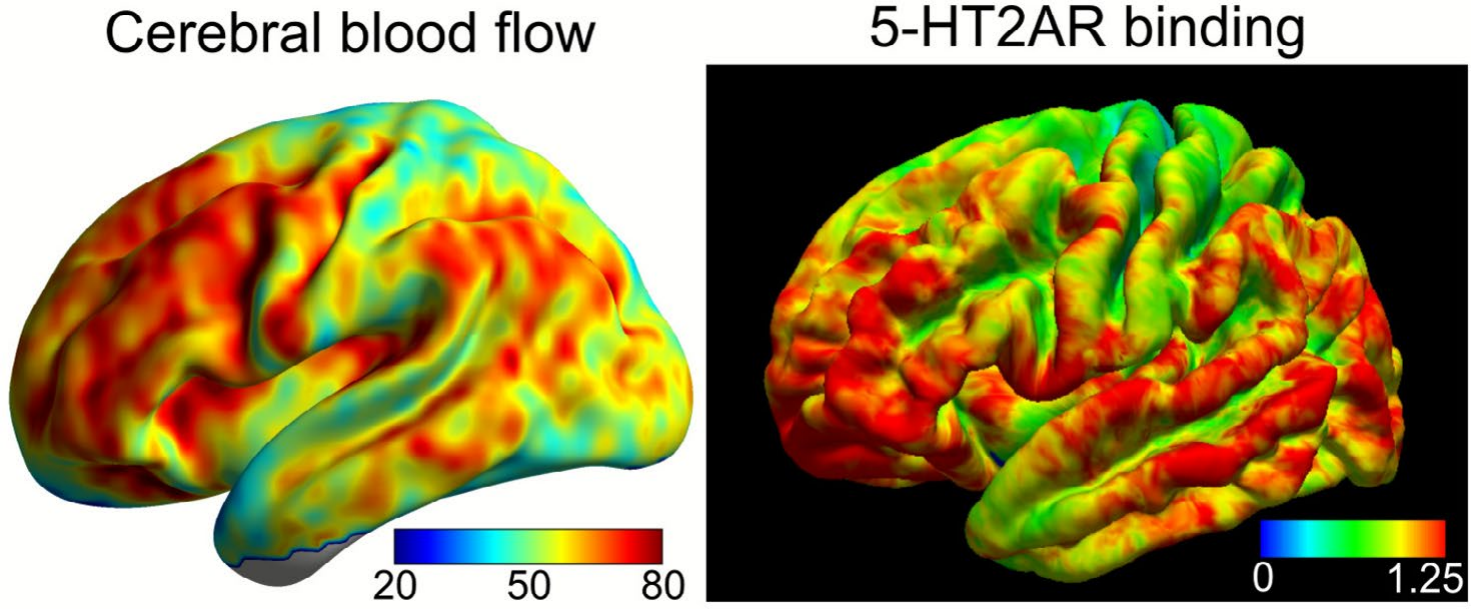
CBF and 5‐HT2AR binding maps. Parametric maps showing population‐level CBF and 5‐HT2AR binding on the left hemisphere surface. Color bars reflect population‐level CBF (ml/100 g/min) and 5‐HT2AR BPND (unitless) values.

For visualization purposes, population‐level surface maps of CBF and 5‐HT2AR binding were calculated (Figure 1). The CBF map was generated by calculating the mean of subject‐specific CBF maps warped into Montreal Neurological Institute (MNI) space using SPM12 and the high‐resolution T1‐weighted structural image. We used BrainNet Viewer (Xia et al. 2013) (https://www.nitrc.org/projects/bnv/) to project this volume onto the surface. The 5‐HT2AR binding map was generated by calculating the median of subject‐specific vertex‐level binding potential maps estimated on the surface with Petsurfer (Greve et al. 2014) and visualized with Freeview v3.0 (http://surfer.nmr.mgh.harvard.edu).

### 2.7 On the Pearson Correlation Coefficient

The Pearson correlation coefficient quantifies the association between two variables based on a set of paired measurements (X, Y). The associated statistical test traditionally assumes that each pair of measurements is independent of each other (and identically distributed). This is a reasonable assumption across participants. For example, if we know the average [11C]Cimbi‐36 BPND value for region A, based on a reference atlas (Beliveau et al. 2017), the region A BPND value of one participant provides no additional information about the region A BPND value for any other participants. However, this assumption is dubious across brain regions. For example, knowing that Participant 1 has a higher than average region A [11C]Cimbi‐36 BPND value may suggest a similarly higher than average region B BPND value (Erritzoe et al. 2010). This between‐region correlation is precisely why repeated‐measures statistical models are commonly applied to brain imaging analyses (e.g., repeated measures ANOVA, linear mixed models, etc.). Therefore, with a two‐level data structure (regional measurements nested into participants), the ordering of operations matters, i.e., the following two procedures are not equivalent: (1) first averaging over participants and then estimating the correlation across regions, which is similar to relating two data types from separate cohorts, or (2) estimating the correlation across participants and averaging those correlations over regions. The former, which relates two data types from separate cohorts, assumes independence across regions whereas the latter, which relates two data types from a single cohort, assumes independence across participants.

This two‐level structure also leads to ambiguity about the definition of the correlation. Indeed, decomposing the residual variability into measurement noise and between‐subject heterogeneity, one can distinguish between the *conditional* correlation (measurement noise only) and the *marginal* correlation (measurement and subject noise). Typically, studies aim to estimate the marginal correlation, e.g., estimating the correlation between 5‐HT2AR binding and CBF in a region over a large, possibly heterogeneous, population. In contrast, with the former, one would focus on a homogenous sub‐group of the population such as young healthy adults with similar genetic state. In practice, researchers want to leverage measurements over multiple regions to make up for the limited number of participants that can be included due to financial and logistic constraints.

### 2.8 Statistical Analyses

All statistical analyses were performed in R (version 4.3.1) (R Core Team 2023). We compared four strategies for estimating the Pearson correlation coefficient between 5‐HT2AR binding and CBF:

- (1.1) Across‐regions strategy (one cohort): all participants have *both* a BPND and CBF measurement; compute mean regional BPND and CBF across all participants; estimate the correlation between BPND and CBF across regions (${\hat{\rho }}_{1.1}$).
- (1.2) Across‐regions strategy (two cohorts): same as Strategy 1.1 except all participants have *either* a BPND or CBF measurement (not both). This is done by splitting the sample in half, using one half (*N* = 12) to calculate the mean regional BPND and the other half (*N* = 12) to calculate the mean regional CBF. This represents what is typically done when correlating data drawn from two separate cohorts (${\hat{\rho }}_{1.2}$).
- (2) Across‐participants strategy: compute the correlation between the observed BPND and CBF across participants, for each region, separately (${\hat{\rho }}_{2}^{R1},{\hat{\rho }}_{2}^{R2},\ldots ,{\hat{\rho }}_{2}^{R18}$). This represents a strategy for correlating two imaging measures drawn from a single cohort.
- (3) Mixed model strategy: the correlation, ${\hat{\rho }}_{3}$, between the observed BPND and CBF across participants for all regions is fit with a single linear mixed model where each participant has 36 measurements, i.e., 18 regions and two modalities (i.e., BPND and CBF). The model includes a mean and variance parameter specific to each region and modality, as well as four correlation parameters. See Supporting Information A1 for additional model details. This strategy is closely related to Strategy 2 but estimates the average marginal correlation instead of region‐specific marginal correlations.

Both Strategies (1) and (2) estimate correlation using the standard implementation of the Pearson correlation coefficient (i.e., *cor.test* from the *stats* package: https://www.rdocumentation.org/packages/stats/versions/3.6.2). Strategy 3 corresponds to a specific residual covariance pattern but can also be fitted as a random effect model when the correlation is positive (see Supporting Information A1). We further explored the influence of sample size on the stability and correspondence of outcomes from Strategy 1.1 and 1.2. To do so, we subsampled our data 10,000 times to generate cohorts of *N* = 4–12 participants and performed Strategy 1.1 (CBF and BPND data from a single cohort) and 1.2 (CBF and BPND from separate cohorts).

Notably, studies combining data from separate cohorts can employ only Strategy 1. Here we leverage that the two imaging data types were acquired in the same cohort to evaluate and compare results from all three strategies. Statistical significance was defined as *p* < 0.05; all reported *p*‐values are not adjusted for multiple comparisons.

### 2.9 Simulation Study

We performed a simulation study to further assess the effect of confounding bias and misinterpretation of inferring across‐regions correlation as evidence for an across‐participants correlation. We assessed the bias of the estimated correlation, coverage of the 95% confidence intervals, and type‐1 error control and statistical power vis‐à‐vis *p* < 0.05 of Strategy 1–3 under five scenarios (A–E) for regional difference in average signal and signal variability (see Table 1 for simulation details). BPND and CBF values were simulated using a multivariate normal distribution with mean, variance, and correlation values estimated based on the observed data, except for the correlation between BPND and CBF in Scenario A–D, which was either set to 0 or to a value larger than the observed. Scenario A and C and E simulate non‐zero correlation between BPND and CBF, whereas Scenario B and D simulate no correlation between BPND and CBF. Correlations for Scenario A and C were selected arbitrarily (marginal/conditional correlation = 0.250/0.500), whereas Scenario E correlation reflects the observed data (marginal/conditional correlation = 0.135/0.057).

**TABLE 1:** Simulation scenarios.

Scenario	Mean		Variance		Correlation			
	Modality specific	Region specific	Modality specific	Region specific	Within modality	Across modalities^a^	Marginal correlation	Conditional correlation
Scenario A	Yes	No	Yes	No	Yes	Yes	0.250	0.500
Scenario B	Yes	Yes	Yes	No	Yes	No	0	0
Scenario C	Yes	Yes	Yes	No	Yes	Yes	0.250	0.500
Scenario D	Yes	No	Yes	Yes	Yes	No	0	0
Scenario E	Yes	Yes	Yes	Yes	Yes	Yes	0.135	0.057

^a^ The marginal and conditional correlations for Scenario E are those estimated by the observed BPND and CBF data.

Each of the above scenarios was simulated 10,000 times using the same structure as our real data, i.e., 18 regions, two measurements (BPND and CBF) and either (1) 24 participants (as in our study) or (2) 1000 participants. For Strategy 1.2, a second dataset of *N* = 24 or 1000 was simulated: mean regional BPND and CBF were calculated from the separate datasets. For Strategy 2, the average of the region‐specific correlations was used and its uncertainty was quantified assuming that the Fisher transformed region‐specific correlations are jointly normally distributed with variance $1/\sqrt{n-3}$ and correlation estimated via the influence function described in Equation (3) of (Devlin et al. 1975). For Strategy 3, the mixed model included a modality‐ and region‐dependent mean but only a modality‐dependent variance (to save computation time), meaning that Strategy 3 was mis‐specified under Scenario D and E. No missing data was considered. A more elaborate description of the data generating mechanism for Simulation Scenario A‐E can be found in Supporting Information A2.

Code used to fit statistical models estimating the correlation coefficients and simulate data can be found here (https://github.com/bozenne/article‐mapping‐fMRI‐PET).

## 3 Results

### 3.1 Across‐Regions Strategy (Strategy 1)

**FIGURE 2:**
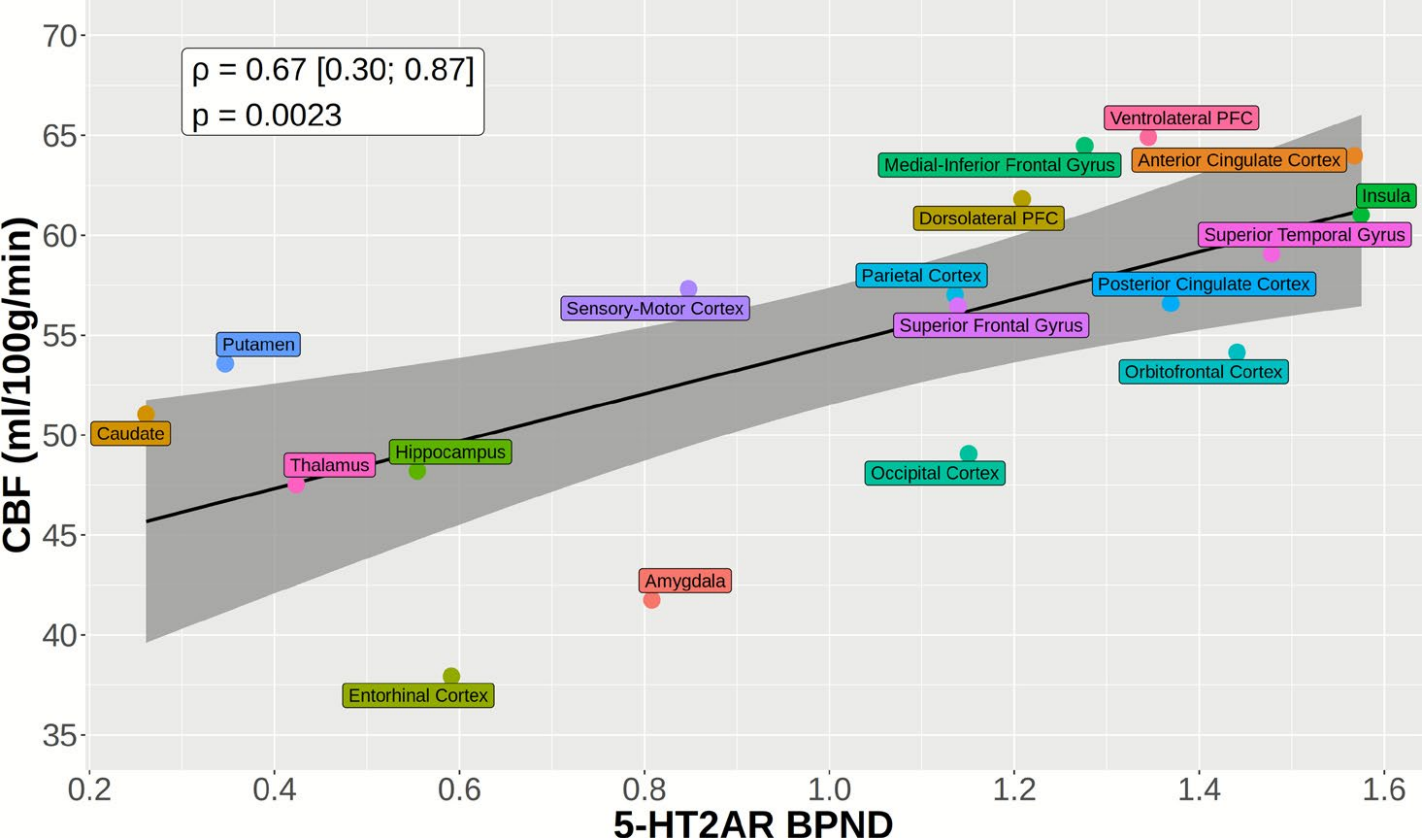
Across‐regions correlation between CBF and 5‐HT2AR BPND based on a single cohort. For each region, the average CBF or 5‐HT2AR BPND value was calculated across participants. The *p*‐value and confidence interval for the Pearson correlation coefficient are estimated without consideration of possible correlation between the regional values from the same modality.

Estimating the across‐regions correlation using data from a single cohort (Strategy 1.1), we observed a statistically significant positive correlation between 5‐HT2AR binding and CBF (${\hat{\rho }}_{1.1}$ [95% CI] = 0.672 [0.298, 0.867]; *p* = 0.0023; Figure 2). We observed similar results when splitting datasets to mimic estimating the across‐regions correlation based on separate cohorts (Strategy 1.2; median ${\hat{\rho }}_{1.2}$ [5% and 95% quantiles] = 0.661 [0.573, 0.741]; median p [5% and 95% quantiles] = 0.003 [< 0.001, 0.013] over 10,000 random splits). However, this was no longer the case when the modality sample size was below 10 (Supporting Information A4). For instance, when considering only four measurements per modality, Strategy 1.2 led to substantially lower correlations (${\hat{\rho }}_{1.2}^{N=4}$ [5% and 95% quantiles] = 0.511 [0.436, 0.587]), whereas Strategy 1.1 exhibited only higher variance (${\hat{\rho }}_{1.1}^{N=4}$ [5% and 95% quantiles] = 0.633 [0.432, 0.777]).

### 3.2 Across‐Participants Strategy (Strategy 2) and Mixed Model Strategy (Strategy 3)

**FIGURE 3:**
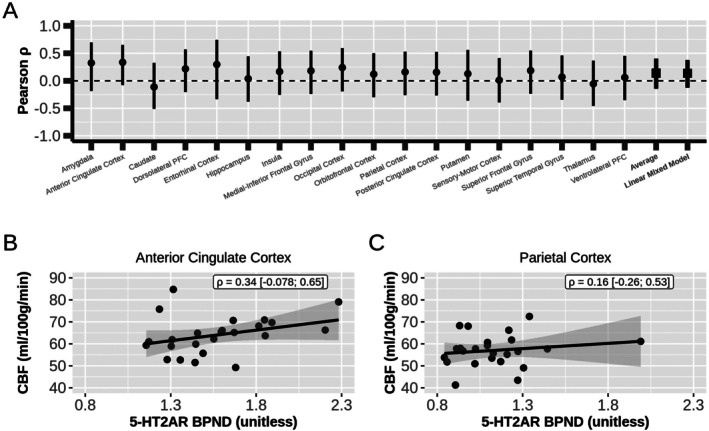
Across‐participants correlations between CBF and 5‐HT2AR BPND. (A) The Pearson correlation coefficient across participants for each region. Points and error bars denote correlation coefficient and 95% confidence interval, respectively. (B) and (C) Regional correlations between 5‐HT2AR binding (BPND) and cerebral blood flow (CBF) for anterior cingulate and parietal cortex, respectively. Estimated Pearson correlation and 95% confidence interval in text box.

Leveraging that the 5‐HT2AR binding and CBF data were acquired in the same cohort, we evaluated the correlation across participants for each region, separately. The estimated correlations between 5‐HT2AR binding and CBF ranged from −0.112 to 0.336 (average ${\hat{\rho }}_{2}$ = 0.140, Figure 3), none were statistically significant (Table 2). A similar result was obtained when using a linear mixed model to estimate the correlation between CBF and BPND across participants with region as a repeated measure (${\hat{\rho }}_{3}$ = 0.138 [−0.100, 0.362]; *p* = 0.26).

**TABLE 2:** Regional correlation coefficients between [11C]Cimbi‐36 BPND and CBF.

Region	Correlation	Lower	Upper	*p*
Amygdala	0.325	−0.185	0.697	0.20
Anterior cingulate cortex	0.336	−0.078	0.651	0.11
Caudate	−0.112	−0.51	0.325	0.62
Dorsolateral prefrontal cortex	0.217	−0.204	0.571	0.31
Entorhinal cortex	0.297	−0.333	0.744	0.35
Hippocampus	0.039	−0.379	0.444	0.86
Insula	0.167	−0.254	0.534	0.44
Medial‐inferior frontal gyrus	0.182	−0.239	0.545	0.39
Occipital cortex	0.240	−0.191	0.594	0.27
Orbitofrontal cortex	0.120	−0.298	0.499	0.58
Parietal cortex	0.160	−0.260	0.529	0.46
Posterior cingulate cortex	0.155	0.265	0.526	0.47
Putamen	0.128	−0.360	0.561	0.61
Sensory‐motor cortex	0.012	−0.393	0.413	0.96
Superior frontal gyrus	0.186	−0.235	0.548	0.38
Superior temporal gyrus	0.069	−0.344	0.460	0.75
Thalamus	−0.055	−0.457	0.365	0.80
Ventrolateral prefrontal cortex	0.060	−0.352	0.453	0.78
Average	0.140	−0.143	0.403	0.33
Linear mixed model (marginal)	0.135	−0.125	0.379	0.31

### 3.3 Simulation Study

Table 3 summarizes the simulation study under each scenario, including a description of the bias in estimated correlation coefficient, coverage (i.e., fraction of times simulated 95% CI includes the true correlation), type‐1 error control (i.e., fraction of times simulated *p* < 0.05 where the null hypothesis is true, relevant for Scenario B and D) and statistical power (i.e., fraction of times simulated *p* < 0.05 where the null hypothesis is false, relevant for Scenario A, C and E). In Scenario A, where there was a true correlation between CBF and BPND and no regional differences, all strategies provided a nearly unbiased estimator (i.e., all biases < 2% different from ground truth) except for Strategy 1.2. Where underlying regional differences in BPND and CBF are introduced (Scenarios B and C), Strategy 1.1 (i.e., across‐regions correlation, single cohort) and 1.2 (i.e., strategy mimicking data drawn from separate cohorts) show substantial bias (Scenario C) and essentially no type‐1 error control in Scenario B (where the null hypothesis is true). Conversely, Strategy 2 and 3 retain low bias (Scenario C: ≤ 2% different from ground truth), type‐1 error control close to nominal level (Scenario B: 4.8%–5.7%), and statistical power that increases when the sample size is increased from *N* = 24 to 1000 participants (Scenario C). Notably, the 100% coverage of Strategy 1.1 and 1.2 in Scenario C was due to wide confidence intervals relative to the bias (width = 0.570, bias = 0.170). Scenario D, which simulated data in the absence of a correlation between BPND and CBF and regional differences in variance but not in mean, showed good coverage and type‐1 error control for all strategies. Scenario E, which simulated data with correlations between BPND and CBF and regional differences in mean and variance, showed a substantial bias for Strategy 1.1 and 1.2 and no coverage, i.e., the correlation coefficient was never well estimated. The model misspecification of Strategy 3 in Scenario E resulted in a negative bias (~10%) and some under‐coverage (90% instead of 95%) for *N* = 1000 participants. Notably, statistical power increases with sample size (*N* = 24 to 1000) for Strategy 2 and 3 under Scenario A, but not for Strategy 1.1 and 1.2. See Supporting Information A3 for a visual representation of simulation scenarios.

**TABLE 3:** Results from simulation study scenarios.

Scenario	Strategy	Region confound	True correlation	Estimated correlation		Bias (%)		Coverage		Type‐1 error		Statistical power	
				*N* = 24	*N* = 1000	*N* = 24	*N* = 1000	*N* = 24	*N* = 1000	*N* = 24	*N* = 1000	*N* = 24	*N* = 1000
A	1.1	No	0.500	0.491	0.491	−1.74	1.83	0.951	0.953	—	—	0.596	0.593
	1.2	No	0.500	0.003	< 0.001	−99.42	−99.95	0.432	0.433	—	—	0.047	0.051
	2	No	0.250	0.244	0.250	−2.27	−0.01	0.942	0.949	—	—	0.490	1
	3	No	0.250	0.253	0.250	1.00	0.06	0.941	0.951	—	—	0.533	1
B	1.1	Yes	0	0.663	0.674	—	—	0	0	1	1	—	—
	1.2	Yes	0	0.663	0.674	—	—	0	0	1	1	—	—
	2	Yes	0	−0.001	< 0.001	—	—	0.943	0.952	0.057	0.048	—	—
	3	Yes	0	−0.001	< 0.001	—	—	0.944	0.952	0.056	0.048	—	—
C	1.1	Yes	0.500	0.668	0.674	33.70	34.80	1	1	—	—	1	1
	1.2	Yes	0.500	0.663	0.674	32.55	34.76	1	1	—	—	1	1
	2	Yes	0.250	0.244	0.250	−2.27	−0.01	0.942	0.949	—	—	0.490	1
	3	Yes	0.250	0.253	0.250	1.00	0.06	0.941	0.951	—	—	0.533	1
D	1.1	Yes	0	0.004	< 0.001	—	—	0.948	0.947	0.051	0.052	—	—
	1.2	Yes	0	−0.002	< 0.001	—	—	0.951	0.947	0.048	0.053	—	—
	2	Yes	0	< 0.001	< 0.001	—	—	0.943	0.950	0.057	0.050	—	—
	3	Yes	0	< 0.001	< 0.001	—	—	0.948	0.953	0.052	0.048	—	—
E	1.1	Yes	0.057	0.663	0.674	1054	1073	0	0	—	—	1	1
	1.2	Yes	0.057	0.662	0.674	1053	1073	0	0	—	—	1	1
	2	Yes	0.135	0.132	0.135	−2.59	0.08	0.941	0.950	—	—	0.177	1
	3	Yes	0.135	0.120	0.123	−11.40	−9.12	0.945	0.902	—	—	0.175	1

*Note:* BPND and CBF are simulated for 18 regions and either 24 or 1000 participants. Scenario A includes correlation between BPND and CBF and no region difference in mean or variance; Scenario B includes no correlation between BPND and CBF and regional variations in mean BPND and CBF; Scenario C includes correlation between BPND and CBF and regional variations in mean BPND and CBF; Scenario D includes no correlation between BPND and CBF and regional variations in mean BPND and CBF based on the observed data; Scenario E includes correlation between BPND and CBF and regional variations in BPND and CBF based on the observed data. Strategy 1.1 (across regions) compares imaging modalities from a single cohort; Strategy 1.2 (across regions) compares imaging modalities from two independent cohorts; Strategy 2 (across participants) estimates the mean of regional correlations between BPND and CBF across regions; Strategy 3 (across participants) estimates the correlation between BPND and CBF within a linear mixed model. See Methods for additional details. Bias is calculated as: (Estimate correlation—True Correlation)/True Correlation. “< 0.001” denotes the absolute value of the estimate is less than 0.001 units from 0.

## 4 Discussion

Recent studies have sought to exploit human brain imaging datasets derived from different cohorts to, e.g., map neuromolecular phenotypes onto brain function or connectivity (Table S1). The results are then often interpreted as evidence for associations between these two measures that generalize to larger populations. Here we analyze a multimodal human brain imaging dataset to provide a counterfactual example of this inference to highlight the pitfalls of incorrectly interpreting correlations estimated across regions, the only available correlation strategy where imaging data are acquired from separate cohorts. We observed a statistically significant positive correlation between 5‐HT2AR BPND, measured with [11C]Cimbi‐36 PET, and CBF, measured with ASL MRI, when modeled across regions, representing an analysis of brain imaging measures acquired in separate cohorts. However, we did not observe evidence for a significant correlation between these two measures for any region, nor in a linear mixed model framework with region as a repeated measure, when modeled across participants, representing the typical analysis where both imaging data are acquired in the same cohort. Our simulation study highlighted that the across‐regions correlation variably and sometimes substantially misestimates the true correlation and can show poor type‐1 error control. Hence, the across‐regions strategy is particularly vulnerable in situations such as where there are regional differences in mean signals, which is common for brain imaging measures. Although our observations do not demonstrate that *all* across‐regions correlations exist in the absence of an across‐participants correlation, they emphasize that these two types of correlations represent two distinct constructs that are not reliably related. This discordance highlights ambiguities around how to interpret an across‐regions correlation and raises concerns about the interpretation of a growing number of studies applying this method to integrate imaging data from two separate cohorts.

A core challenge with the across‐regions analysis strategy can be understood by considering the underlying assumptions of the Pearson correlation coefficient, ρ. This can be effectively estimated based on *independent* replicates of two variables, X and Y, that are not confounded. The across‐regions strategy, e.g., Strategy 1, grossly violates this assumption in many (if not all) multimodal human brain imaging analyses. For instance, regional PET measurements are typically highly correlated within a participant (Erritzoe et al. 2010) and both PET and fMRI show substantial regional variations in population mean value (Figure 2). Our simulations show that the correlation estimated by Strategy 1 (across regions) can be very sensitive to regional mean differences and variably biased with respect to the correlation structure across participants. The 5‐HT2AR and CBF data used here highlight this confound: there are regional differences in both 5‐HT2AR and CBF and they have a similar regional rank order structure. Subsequently, for our observed dataset, we observed a significant correlation across regions (Strategy 1), whereas the regional correlations across participants (Strategy 2 and 3) were substantially lower and not statistically significant.

Our simulation results further suggest the breadth of circumstances where these biases affect estimated correlations. In Scenario B there is no underlying correlation between BPND and CBF; yet a strong correlation coefficient is ubiquitously estimated by the across‐regions strategy. Subsequently, the type‐1 error rate, which should be 5%, can climb to 100%. In Scenario C, where there is a true underlying correlation between BPND and CBF, the across‐regions strategy (Strategy 1) consistently misestimates this correlation. Although Strategy 1 correctly estimates a null effect in Scenario D, it has a substantial bias and no coverage in Scenario E, where the regional 5‐HT2AR binding and CBF differences are equivalent. The erratic bias of Strategy 1 across scenarios highlights that it is highly susceptible to error, irrespective of whether there is a true underlying correlation. Alternative strategies are not without problems, which is illustrated in Scenario E where the variance was incorrectly modeled by the linear mixed model. Specifying the variance and correlation via a linear mixed model can be challenging, especially with fewer participants compared to the number of regions. Nevertheless, a reasonable model structure showed some robustness to misspecification, particularly vis‐à‐vis the dramatic effect of ignoring confounding by region.

Notably, from our simulated data, we show that the across‐regions strategy estimates a high correlation coefficient in the presence of regionally variable estimates, irrespective of whether there is (Simulation Scenario C) or is not (Simulation Scenario B) a true underlying correlation between the two estimates. Essentially, this means that it is very difficult, or perhaps not even possible, to dissociate the correlation due to this confound and true correlation between measures across participants. This bias is particularly problematic vis‐à‐vis a commonly applied spatial permutation framework (Alexander‐Bloch et al. 2018). Like other permutation tests, this approach controls the type‐1 error (assuming exchangeability), whereas we show that the correlation estimate itself is variably and sometimes substantially biased, limiting its interpretability, irrespective of the type‐1 error control. It is not currently clear to us how one can estimate this confound between two imaging measures without measuring both in a single cohort, which limits the application of datasets from different cohorts. Indeed, controlling for confounding typically requires individual measurement of the confounder, exposure, and the outcome. It worth remembering that a linear model regressing, e.g., BPND on CBF (or vice versa), estimates a slope parameter that is the Pearson correlation rescaled by the standard deviation of the outcome and the exposure, i.e., $\beta =\rho \frac{\sigma_{y}}{\sigma_{x}}$, and the corresponding Wald test statistic can be shown to be a function of the Pearson correlation and the sample size, i.e., $t_{\beta }=\frac{\overset{`}{\beta }}{\sigma_{\overset{`}{\beta }}}=\frac{\rho }{\sqrt{\left(1-\rho^{2}\right)/\left(n-2\right)}}$. Put another way, concerns about confounding when fitting a linear regression also apply to estimating correlation coefficients.

Another perspective that highlights the limitation of exchanging across‐regions correlations for evidence of across‐participants correlations can be seen through the variance–covariance matrix that emerges from a linear mixed model fit when data are from a single cohort (see Supporting Information A1). From this model we can estimate the correlation between BPND and CBF from the same region ($r_{\text{region}}$). By contrast, the across‐regions correlation is not directly estimated within the variance–covariance matrix, suggesting that it captures a correlation not straight‐forwardly aligned with how correlation is typically understood by practitioners nor estimated when data are drawn from a single cohort.

An observation from the simulation study that highlights the conceptual peculiarity of the across‐regions correlation can be seen in the statistical power. In Simulation Scenario A, where a true correlation exists, the statistical power of the across‐regions strategies (Strategy 1.1 and 1.2) is unchanged increasing from *N* = 24 to 1000 participants. That the number of participants observed is essentially irrelevant to the uncertainty of the correlation estimate, and subsequently the *p*‐value, speaks to how this does not reflect a correlation estimate that is generalizable to broader populations. Conversely, and similarly counterintuitively, increasing the number of regions, i.e., parcellating the brain into smaller units, *would* increase the statistical power precisely because it is a correlation across regions.

Here we use an available multimodal brain imaging dataset to highlight the limitations of this across‐regions analysis strategy. The simulation study results reinforce that these pitfalls are not unique to the idiosyncratic features of the current empirical dataset: (1) although CBF and [11C]Cimbi‐36 PET likely have their own susceptibility “fingerprint”, the underlying statistical problem generalizes to any pair of spatial maps, such as BOLD fMRI task activation and connectivity; (2) sample size, our simulation studies show that across‐regions correlations are counterintuitively largely independent of participant sample size; (3) due to the nature of the across‐regions correlation, increasing the number of regions will generally amplify the type‐1 error, which we show in our simulation study is already highly vulnerable with only 18 regions; and (4) statistical model, here we applied a simple linear regression across regions (Strategy 1), but the same inferential misinterpretation extends to multivariate estimations (e.g., canonical correlation analysis) of correlation/association.

Our observations do not speak to all applications of atlas parcellations derived from independent cohorts. For example, we see no concern with a strategy that estimates the mean fMRI task response within regions of interest defined by having high 5‐HT2AR levels. The use of the atlas, in and of itself, does not clearly bias the fMRI estimate nor bias its relation to another measure. However, it does not follow that the fMRI task response, or its correlation with another measure, is somehow related to 5‐HT2AR because of how the regions were defined.

One may argue that because multimodal studies are sometimes prohibitively expensive or impractical, estimating across‐regions correlations from separate cohorts is “better than nothing.” The absence of a mathematical link between across‐regions and across‐participants correlations and the erratic divergence from known correlations that we observed across our empirical data and simulation studies suggests these results are unpredictably misleading and therefore not clearly useful in the absence of multimodal data.

In summary, we present a counterfactual example that challenges the increasingly published interpretation that an across‐regions correlation between imaging datasets from separate cohorts constitutes evidence for a relation between the two imaging measures that generalizes to broader populations. In addition to misleading the assessment of molecular mechanisms associated with functional brain imaging phenotypes, the misinterpretation of these across‐regions correlations could facilitate, e.g., human pharmacological studies focusing on drug targets erroneously determined to be related to an imaging measure or clinical phenotype. We thus recommend researchers and associated stakeholders be mindful of these pitfalls.

## Funding

The data were collected as part of NeuroPharm (np.nru.dk) and supported by the following sources: Innovation Fund Denmark (grant number: 4108‐00004B), Independent Research Fund Denmark (grant number: 6110‐00518B), Marie‐Curie‐NEUROMODEL (grant number: 746850).

## Conflicts of Interest

The authors declare no conflicts of interest.

## Supporting information


**Figure S1:** Scatterplot across regions of a simulated dataset for Scenario A, B, and C, with a different panel per participant (first four columns) or the average over participants (fifth column).
**Figure S2:** Same scatterplots as in Figure S1, except that observations have been centered and scaled using region‐specific means and standard deviations.
**Figure S3:** Scatterplot across participants of the same simulated dataset as in Figure S1, except here with a different panel per region. The boxplot (rightmost column) displays the region‐specific correlations which are closely related to the slopes shown in the scatterplots.
**Figure S4:** Boxplots showing estimated spatial correlations between BPND and CBF across participant subsamples.
**Table S1:** Example studies that evaluate across region correlations or estimations with spatial brain maps from two separate cohorts for the purpose of inferring generalizable relations between functional and molecular maps. Additional citation information can be found in Supplementary References.

## Data Availability

Code for analyses can be found here: https://github.com/bozenne/article‐mapping‐fMRI‐PET. Individual participant data can be made available with an appropriate data sharing agreement; please contact the corresponding author for more information.
