# Supplementary material for: Discordance Between Spatial and Population Correlations From Human Brain Imaging Data
Source: Hum Brain Mapp. 2025 Nov 26;46(17):e70421. doi: 10.1002/hbm.70421 (PMC12648428; doi:10.1002/hbm.70421)
Supplement: Supplementary file 1 — Figure S1: Scatterplot across regions of a simulated dataset for Scenario A, B, and C, with a different panel per participant (first four columns) or the average over participants (fifth column). Figure S2: Same scatterplots as in Figure S1, except that observations have been centered and scaled using region‐specific means and standard deviations. Figure S3: Scatterplot across participants of the same simulated dataset as in Figure S1, except here with a different panel per region. The boxplot (rightmost column) displays the region‐specific correlations which are closely related to the slopes shown in the scatterplots. Figure S4: Boxplots showing estimated spatial correlations between BPND and CBF across participant subsamples. Table S1: Example studies that evaluate across region correlations or estimations with spatial brain maps from two separate cohorts for the purpose of inferring generalizable relations between functional and molecular maps. Additional citation information can be found in Supplementary References. [file HBM-46-e70421-s001.docx]

**SUPPLEMENTARY MATERIAL**

**Discordance between spatial and population correlations from human brain imaging data**

Patrick M. Fisher^1,2,*^, Kristian Larsen^1,3^, Pontus Plavén-Sigray^1,4^, Gitte M. Knudsen^1,3^, Brice Ozenne^1,5^

^1^Neurobiology Research Unit, Copenhagen University Hospital Rigshospitalet, Copenhagen, Denmark

^2^Department of Drug Design and Pharmacology, University of Copenhagen, Copenhagen, Denmark

^3^Department of Clinical Medicine, University of Copenhagen, Copenhagen, Denmark

^4^Department of Clinical Neuroscience, Centre for Psychiatry Research, Karolinska Institutet and Stockholm Health Care Services, Region Stockholm, Stockholm, Sweden

^5^Section on Biostatistics, Department of Public Health, University of Copenhagen, Copenhagen, Denmark

***Corresponding author**

Patrick M. Fisher, PhD

Neurobiology Research Unit

Rigshospitalet 8057

Copenhagen, Denmark

patrick@nru.dk

**Supplementary Table 1.** Example studies that evaluate across region correlations or estimations with spatial brain maps from two separate cohorts for the purpose of inferring generalizable relations between functional and molecular maps. Additional citation information can be found in Supplementary References.

**Supplementary Material A1**. Specification of the linear mixed model

The proposed linear mixed model (Strategy 3) uses four correlations parameters to model the within and between modality correlation:

- $r_{BPND}$: correlation between BPND measurements from different regions
- $r_{CBF}$: correlation between CBF measurements from different regions
- $r_{region}$: correlation between a BPND and a CBF measurement from the same region
- $r_{id}$: correlation between a BPND and a CBF measurement from different regions

The resulting correlation matrix is shown below, considering three example regions (thalamus, putamen, caudate):

$$Cor\left( \begin{matrix} \mathrm{thalamus}_{\mathrm{BPND}} \\ \mathrm{putamen}_{BPND} \\ \mathrm{caudate}_{BPND} \\ \mathrm{thalamus}_{\mathrm{CBF}} \\ \mathrm{putamen}_{\mathrm{CBF}} \\ \mathrm{caudate}_{\mathrm{CBF}} \end{matrix} \right)=\left[ \begin{matrix} 1 & r_{BPND} & r_{BPND} & r_{region} & r_{id} & r_{id} \\ r_{BPND} & 1 & r_{BPND} & r_{id} & r_{region} & r_{id} \\ r_{BPND} & r_{BPND} & 1 & r_{id} & r_{id} & r_{region} \\ r_{region} & r_{id} & r_{id} & 1 & r_{CBF} & r_{CBF} \\ r_{id} & r_{region} & r_{id} & r_{CBF} & 1 & r_{CBF} \\ r_{id} & r_{id} & r_{region} & r_{CBF} & r_{CBF} & 1 \end{matrix} \right]$$

Based on the Restricted Maximum Likelihood estimates of the correlation parameters, the correlation of interest was estimated as $\hat{\rho}=\frac{\hat{r}_{region}-\hat{r}_{id}}{\sqrt{(1-\hat{r}_{BPND})(1-\hat{r}_{CBF})}}$. This formula has been derived from an alternative, and more restrictive, formulation of the model using random effects, where for region $r$:

$${BPND}_{r}= \alpha_{r}+u_{i}+\delta_{i}$$

$${CBF}_{r}= \beta_{r}+v_{i}+\varepsilon_{i}$$

$\alpha$ and $\beta$ denote the region-specific intercepts, $u$ and $v$ denote the participant- and modality-specific random effects, and $\delta$ and $\varepsilon$, the residuals. Random effects and residuals are modeled to be independent from each other:

$$\left[ \begin{matrix} u \\ v \\ \delta\\ \varepsilon\end{matrix} \right]\mathcal{\sim N}\left( \left[ \begin{matrix} 0 \\ 0 \\ 0 \\ 0 \end{matrix} \right],\left[ \begin{matrix} \tau_{BPND} & \tau_{BPND,CBF} & 0 & 0 \\ \tau_{BPND,CBF} & \tau_{CBF} & 0 & 0 \\ 0 & 0 & \sigma_{BPND}^{2} & \sigma_{BPND,CBF}^{2} \\ 0 & 0 & \sigma_{BPND,CBF}^{2} & \sigma_{CBF}^{2} \end{matrix} \right] \right)$$

It can then be shown when $r_{BPND}>0$, $r_{CBF}>0$, $r_{region}>r_{id}>0$ and assuming that the variance is not region dependent, but may be modality dependent, the proposed mixed model and the random effect models are equivalent and $Cor\left( u_{i}+\delta_{i},v_{i}+\varepsilon_{i} \right)=\frac{\sigma_{BPND,CBF}^{2}+ \tau_{BPND,CBF}}{\sqrt{(\sigma_{BPND}^{2}+\tau_{BPND})(\sigma_{CBF}^{2}+\tau_{CBF})}}=\frac{r_{region}-r_{id}}{\sqrt{(1-r_{BPND})(1-r_{CBF})}}$

**Supplementary Material A2**. Simulation study data generating mechanism

The data were simulated considering 18 regions and using a multivariate normal distribution parametrized according to the estimated parameters of a linear mixed model with modality and region dependent mean, modality dependent variance (Scenario A, B, C) or modality and region dependent variance (Scenario D, E), and the correlation structure described in Supplementary Material A1. This leads to the following values:

- Mean: Scenario A and D: mean BPND of 1.027 and mean fMRI of 54.772; Scenario B, C, E: region dependent means, BPND: 0.242 to 1.575; and CBF: 37.933 to 64.914.
- Standard deviation: Scenario A, B; C: standard deviation BPND of 0.255 and standard deviation CBF of 9.270; Scenario D, E: region dependent standard deviations, BPND: 0.105 to 1.399; and CBF: 6.355 to 16.998.
- Within-modality correlation: Scenario A, B, C: within-modality correlation, $r_{BPND}=$0.638 and $r_{CBF}=$0.481; Scenario D, E: $r_{BPND}=$ 0.684 and $r_{CBF}=$ 0.554.
- Cross-modality correlation: same regions correlation, Scenario A and C: $r_{region}=$0.500; Scenario B and D: $r_{region}=$ 0; Scenario E: $r_{region}=$ 0.135; different regions correlation, Scenario A and C: $r_{id}=$0.392 (leading to a conditional correlation of 0.250); Scenario B and D: $r_{id}=$ 0; Scenario E: $r_{id}=$ 0.113 (leading to a conditional correlation of 0.0574).

**Supplementary Material A3**. Strategy 1 vs. Strategy 2 on a simulated dataset

To further illustrate the difference between Strategy 1 and 2 and how regional mean differences can affect Strategy 1, consider three datasets containing 24 participants each, simulated for Scenario A, B, and C, with the same starting value the random number generator (seed 12) corresponding to. The seed was chosen such that the estimated correlation with Strategy 2 approximately matched the marginal correlation defined by each scenario.

Supplementary Figure 1 displays the individual (first four columns) as well as the mean regional 5-HT2AR binding and CBF across all participants (fifth column). The correlation estimated by Strategy 1 is closely related to the regression slope shown in the fifth column. Visually, the data generated under Scenario B and C appear similar as most of the variation is driven by the large differences in regional mean, e.g., most patients have large BPND and CBF values in ventrolateral PFC and low values in caudate.

Supplementary Figure 2 displays the same data but normalized for each region, i.e., subtract the regional mean and divide by the regional standard deviation (across participants). This normalization does not have a large effect on Scenario A, but substantially affects Scenario B: the absence of correlation between BPND and CBF is much more obvious after removing regional effects.

Supplementary Figure 3 displays the same dataset, instead focusing on a subset of regions (the colored points in Supplementary Figure 1 match the points with numbers in Supplementary Figure 3). The regional difference in mean in Scenario B and C us apparent from the graphical display, yet does not affect the estimated Pearson correlation.


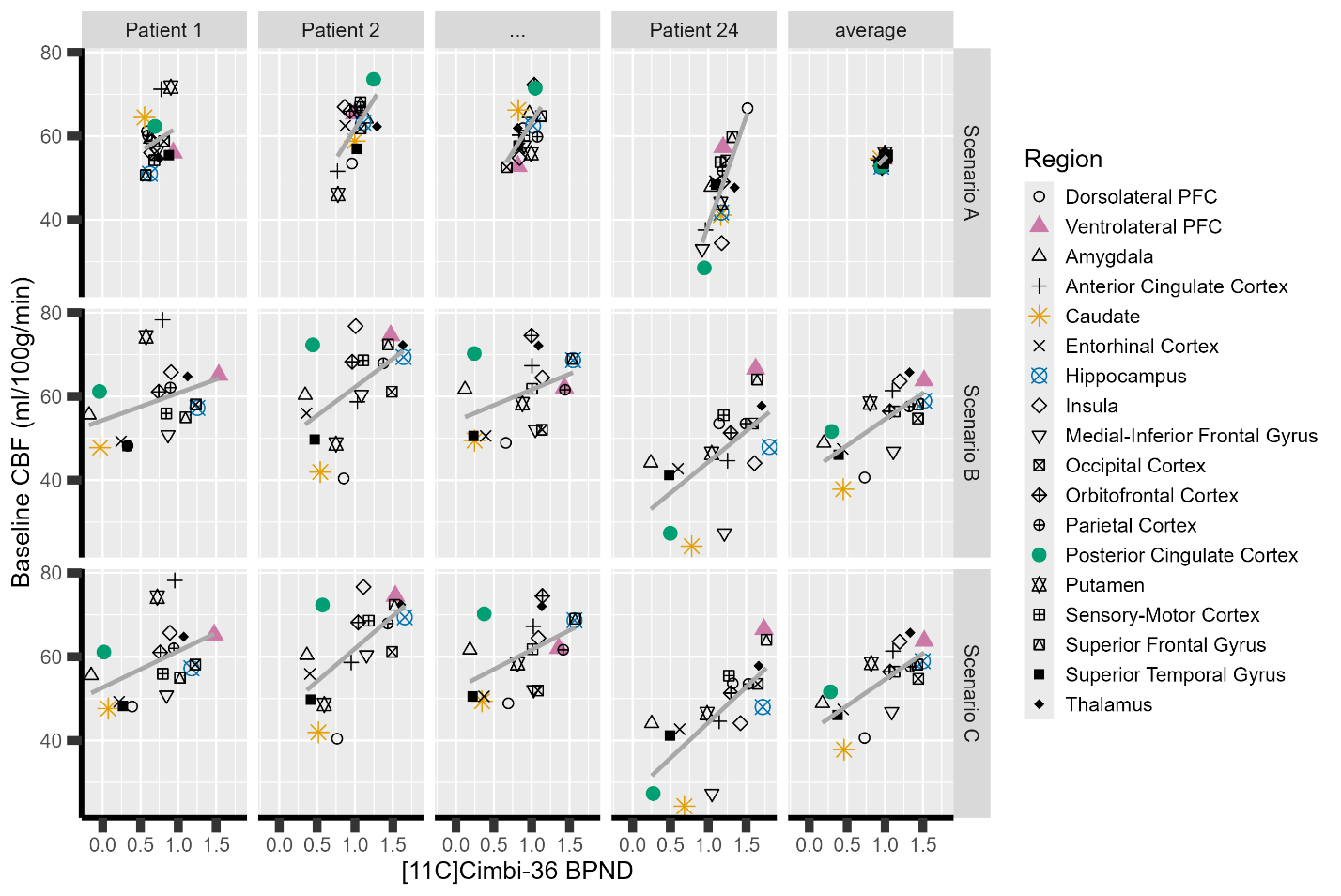


Supplementary Figure 1. Scatterplot across regions of a simulated dataset for Scenario A, B, and C, with a different panel per participant (first four columns) or the average over participants (fifth column).


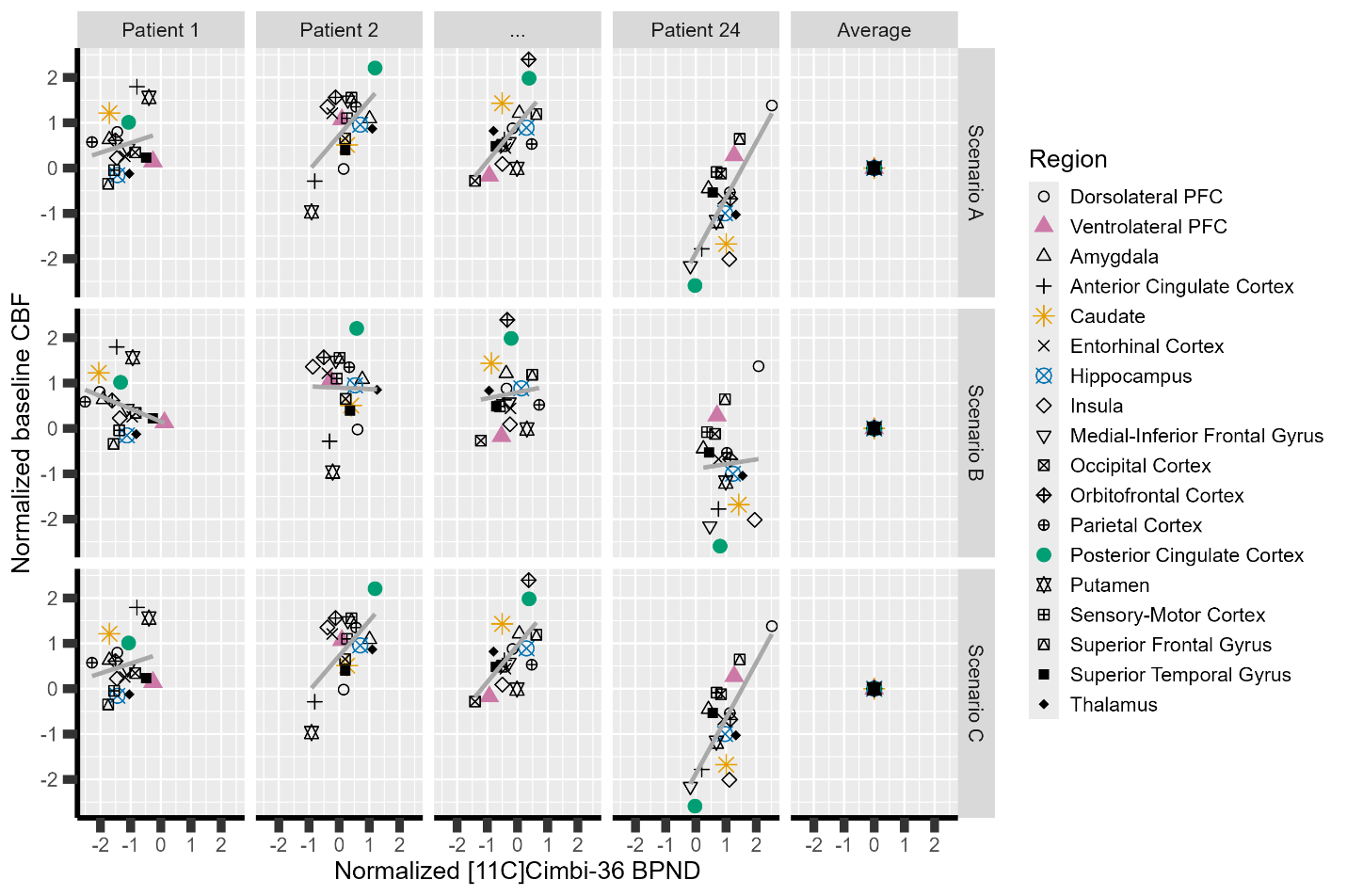


Supplementary Figure 2. Same scatterplots as in Supplementary Figure 1, except that observations have been centered and scaled using region-specific means and standard deviations.


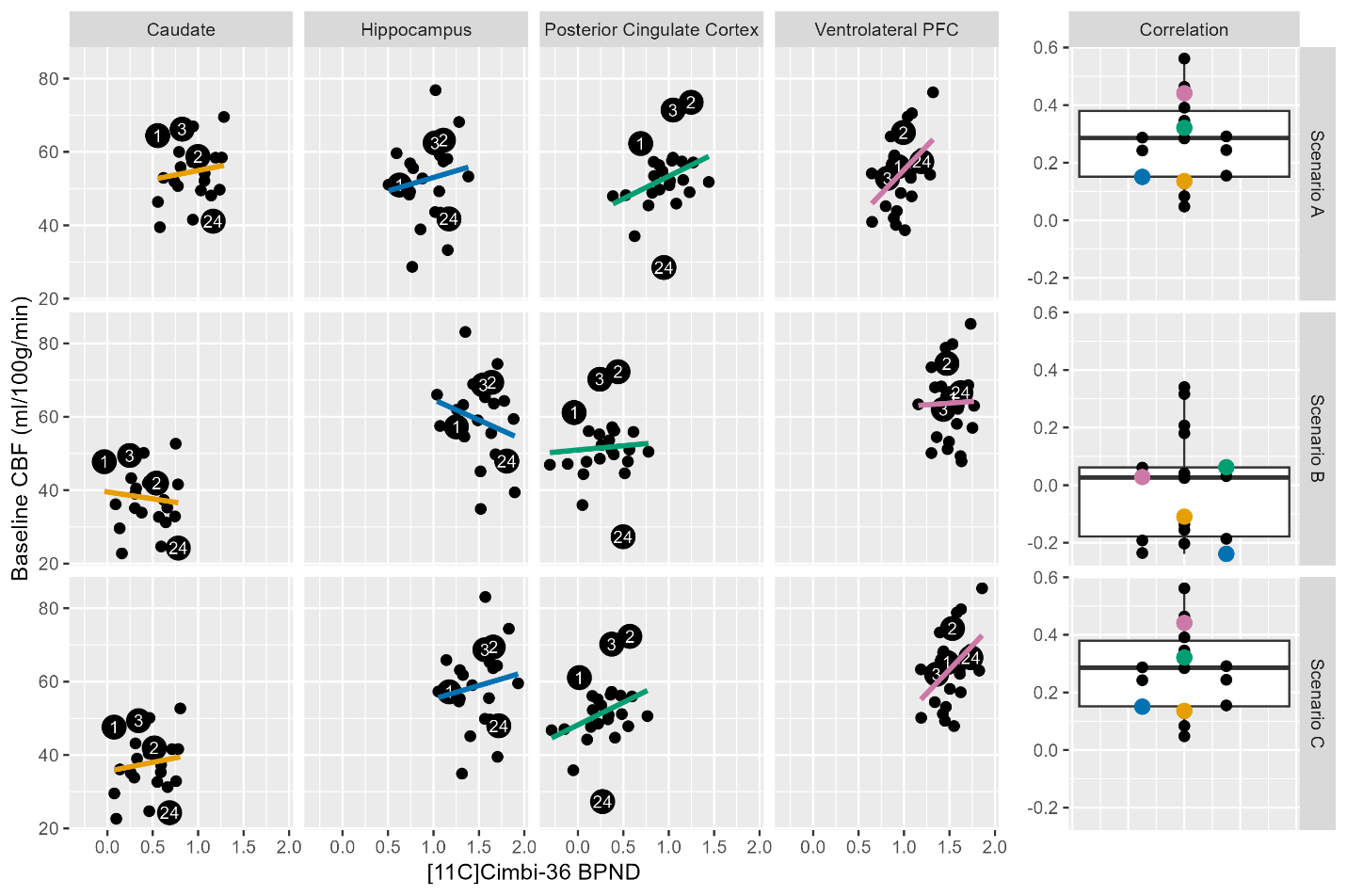


Supplementary Figure 3. Scatterplot across participants of the same simulated dataset as in Supplementary Figure 1, except here with a different panel per region. The boxplot (rightmost column) displays the region-specific correlations which are closely related to the slopes shown in the scatterplots.

**Supplementary Material A4**. Strategy 1.1 vs. Strategy 1.2 in subsamples

To illustrate the behavior of the correlation estimators in small samples for Strategy 1.1 and 1.2, we drew random subsamples from the observed BPND and CBF data:

- Strategy 1.1 (single cohort): draw $n$ participants, where $n$ is varied between four and 12, and
- Strategy 1.2 (independent cohorts): draw $2n$ participants where $n$ was varied between four and 12, set CBF values to missing for the first $n$ subjects and the BPND values to missing for the last $n$ participants.

This ensured that $n$ values were available for each modality regardless of the strategy. For each sample size, subsampling was repeated 10000 times, and the results were displayed as boxplot in the figure below:


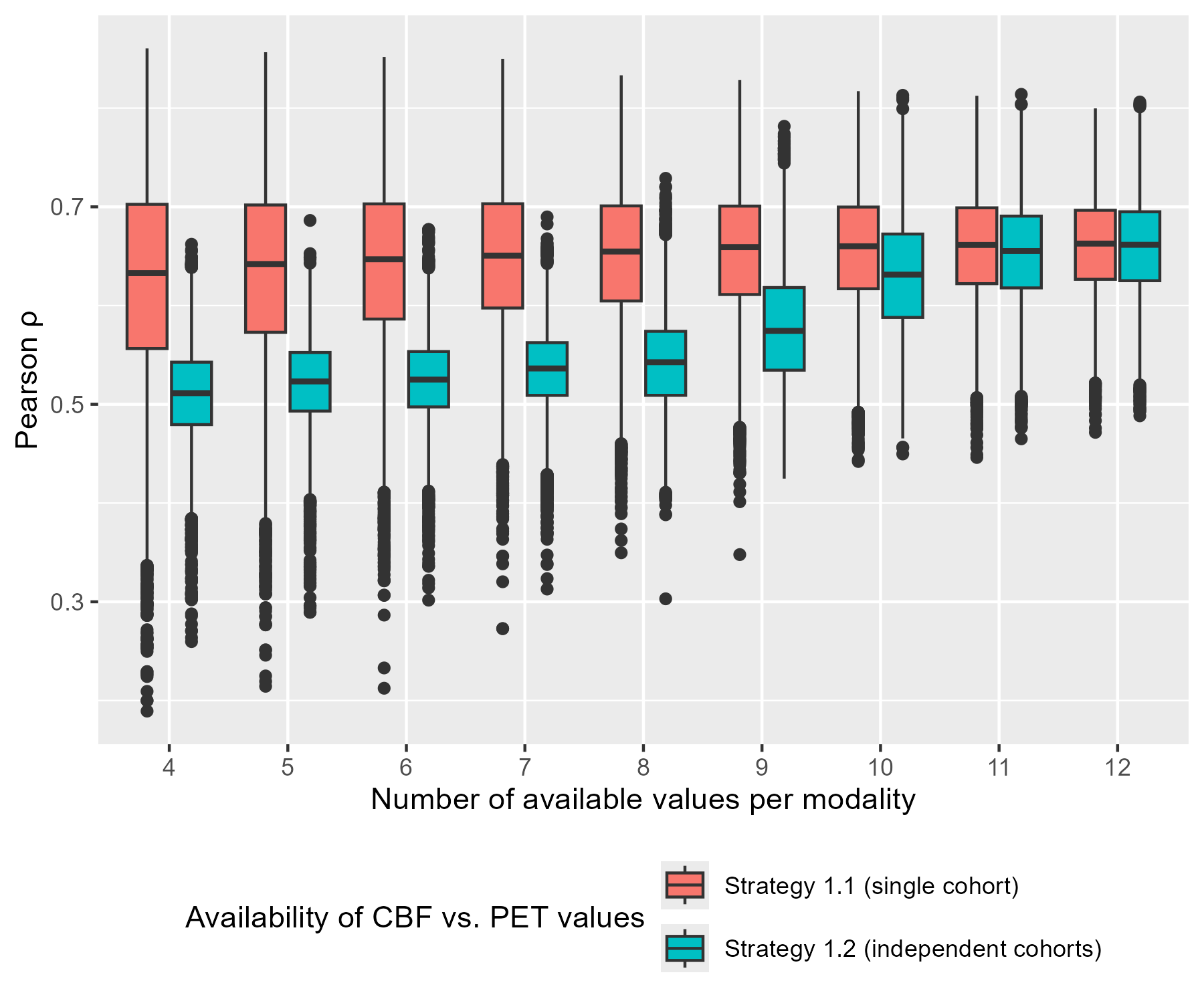
Supplementary Figure 4. Boxplots showing estimated spatial correlations between BPND and CBF across participant subsamples.

Lowering the sample size only appears to increase the variance of the Strategy 1.1. estimator. In contrast, the Strategy 1.2 estimator underestimates the spatial correlation in small samples. The effect becomes noticeable at sample sizes below $n$ = 10 (unique participants per modality). It worth noting that both estimators lead to substantially different results from the across participants correlation found by Strategy 2 or 3 (average $\hat{\rho}_{2}$ = 0.140, $\hat{\rho}_{3}$ = 0.138), based on the full sample.

To explain the apparent small sample bias of strategy 1.2, consider a simplified region-specific model where the BPND (denoted $X$) and CBF (denoted $Y$) values for each participant, $i$, in region, $r$, follow:

$$X_{i,r}= \mu_{X_{r}}+ \sigma_{X_{r}}\varepsilon_{i_{X}}$$

$$Y_{i,r}= \mu_{Y_{r}}+ \sigma_{Y_{r}}\varepsilon_{i_{Y}}$$

The model involves region-specific means, $\mu,$ and variance, $\sigma$, that are jointly normally distributed, i.e., the residuals, $\varepsilon$, are jointly normally distributed and possibly correlated. Strategy 1 first averages over participants, which retains the mean structure but modifies the variance-covariance, essentially rescaling the variance by $\sqrt{n}$, where $n$ is the number of participants:


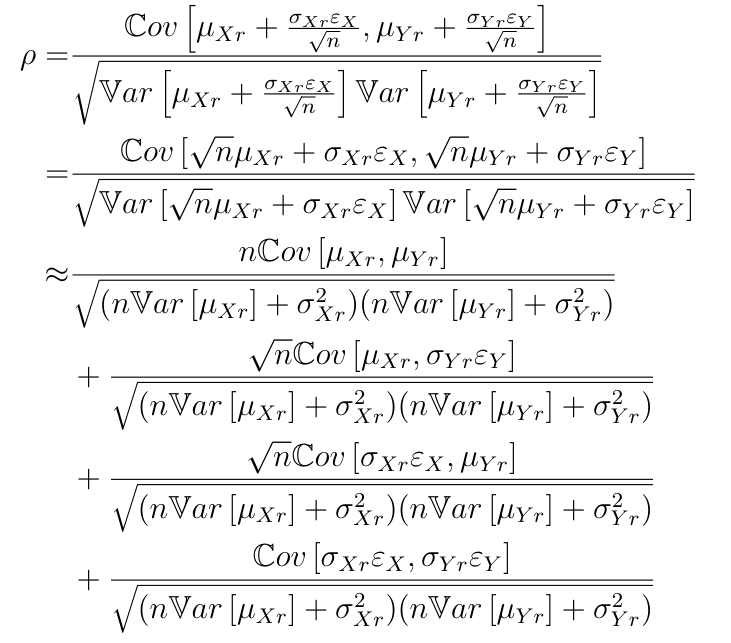


Under Scenario A the mean is constant across regions (an unusual characteristic of real data), so the first three terms are zero and only the last term contributes. For our real data, Scenarios B-E, and in nearly all other real cases, the regional mean covariance bias term dominates with a sufficiently large sample, limiting the influence of the two middle terms. Strategy 1.1 and 1.2 can diverge via the last term, which is also zero in Strategy 1.2, i.e., BPND and CBF are drawn from two independent cohorts, but is non-zero in Strategy 1.1, i.e., BPND and CBF are drawn from the same cohort. The last term is relatively more prominent in small samples because it does not have $n$ or $\sqrt{n}$ in the numerator, providing an explanation for the divergence between Strategy 1.1 and 1.2 in small samples observable in Supplementary Figure 4.

**Supplementary References**

Aqil, M., Knapen, T., & Dumoulin, S. O. (2024). Computational model links normalization to chemoarchitecture in the human visual system. *Science Advances*, *10*(1), 1–11. https://doi.org/10.1126/sciadv.adj6102

Avram, M., Fortea, L., Wollner, L., Coenen, R., Korda, A., Rogg, H., … Borgwardt, S. (2024). Large-scale brain connectivity changes following the administration of lysergic acid diethylamide, d-amphetamine, and 3,4-methylenedioxyamphetamine. *Molecular Psychiatry*, (January). https://doi.org/10.1038/s41380-024-02734-y

Bazinet, V., Hansen, J. Y., & Misic, B. (2023). Towards a biologically annotated brain connectome. *Nature Reviews Neuroscience*, *24*(12), 747–760. https://doi.org/10.1038/s41583-023-00752-3

Bazinet, V., Hansen, J. Y., Vos de Wael, R., Bernhardt, B. C., van den Heuvel, M. P., & Misic, B. (2023). Assortative mixing in micro-architecturally annotated brain connectomes. *Nature Communications*, *14*(1), 2850. https://doi.org/10.1038/s41467-023-38585-4

Betzel, R. F., Puxeddu, M. G., Seguin, C., Bazinet, V., Luppi, A., Podschun, A., … Parkes, L. (2024, February 28). Controlling the human connectome with spatially diffuse input signals. https://doi.org/10.1101/2024.02.27.581006

Boucherie, D. E., Reneman, L., Booij, J., Martins, D., Dipasquale, O., & Schrantee, A. (2023). Modulation of functional networks related to the serotonin neurotransmitter system by citalopram: Evidence from a multimodal neuroimaging study. *Journal of Psychopharmacology*, *37*(12), 1209–1217. https://doi.org/10.1177/02698811231211154

Burt, J. B., Preller, K. H., Demirtas, M., Ji, J. L., Krystal, J. H., Vollenweider, F. X., … Murray, J. D. (2021). Transcriptomics-informed large-scale cortical model captures topography of pharmacological neuroimaging effects of LSD. *ELife*, *10*. https://doi.org/10.7554/eLife.69320

Cercignani, M., Dipasquale, O., Bogdan, I., Carandini, T., Scott, J., Rashid, W., … Bozzali, M. (2021). Cognitive fatigue in multiple sclerosis is associated with alterations in the functional connectivity of monoamine circuits. *Brain Communications*, *3*(2). https://doi.org/10.1093/braincomms/fcab023

Dipasquale, O., Cohen, A., Martins, D., Zelaya, F., Turkheimer, F., Veronese, M., … Wang, Y. (2023). Molecular-enriched functional connectivity in the human brain using multiband multi-echo simultaneous ASL/BOLD fMRI. *Scientific Reports*, *13*(1), 11751. https://doi.org/10.1038/s41598-023-38573-0

Dukart, J., Holiga, Š., Chatham, C., Hawkins, P., Forsyth, A., McMillan, R., … Sambataro, F. (2018). Cerebral blood flow predicts differential neurotransmitter activity. *Scientific Reports*, *8*(1), 4074. https://doi.org/10.1038/s41598-018-22444-0

Froudist-Walsh, S., Bliss, D. P., Ding, X., Rapan, L., Niu, M., Knoblauch, K., … Wang, X.-J. (2021). A dopamine gradient controls access to distributed working memory in the large-scale monkey cortex. *Neuron*, *109*(21), 3500-3520.e13. https://doi.org/10.1016/j.neuron.2021.08.024

Goulas, A., Changeux, J.-P., Wagstyl, K., Amunts, K., Palomero-Gallagher, N., & Hilgetag, C. C. (2021). The natural axis of transmitter receptor distribution in the human cerebral cortex. *Proceedings of the National Academy of Sciences*, *118*(3). https://doi.org/10.1073/pnas.2020574118

Hansen, J. Y., Cauzzo, S., Singh, K., García-Gomar, M. G., Shine, J. M., Bianciardi, M., & Misic, B. (2024). Integrating brainstem and cortical functional architectures. *Nature Neuroscience*, *27*(12), 2500–2511. https://doi.org/10.1038/s41593-024-01787-0

Hansen, J. Y., Markello, R. D., Tuominen, L., Nørgaard, M., Kuzmin, E., Palomero-Gallagher, N., … Misic, B. (2022). Correspondence between gene expression and neurotransmitter receptor and transporter density in the human brain. *NeuroImage*, *264*, 119671. https://doi.org/10.1016/j.neuroimage.2022.119671

Hansen, J. Y., Shafiei, G., Markello, R. D., Smart, K., Cox, S. M. L., Nørgaard, M., … Misic, B. (2022). Mapping neurotransmitter systems to the structural and functional organization of the human neocortex. *Nature Neuroscience*, *25*(11), 1569–1581. https://doi.org/10.1038/s41593-022-01186-3

Hansen, J. Y., Shafiei, G., Vogel, J. W., Smart, K., Bearden, C. E., Hoogman, M., … Misic, B. (2022). Local molecular and global connectomic contributions to cross-disorder cortical abnormalities. *Nature Communications*, *13*(1), 4682. https://doi.org/10.1038/s41467-022-32420-y

He, K., Hua, Q., Li, Q., Zhang, Y., Yao, X., Yang, Y., … Wang, K. (2023). Abnormal interhemispheric functional cooperation in schizophrenia follows the neurotransmitter profiles. *Journal of Psychiatry and Neuroscience*, *48*(6), E452–E460. https://doi.org/10.1503/jpn.230037

Lawn, T., Dipasquale, O., Vamvakas, A., Tsougos, I., Mehta, M. A., & Howard, M. A. (2022). Differential contributions of serotonergic and dopaminergic functional connectivity to the phenomenology of LSD. *Psychopharmacology*, *239*(6), 1797–1808. https://doi.org/10.1007/s00213-022-06117-5

Lawn, T., Howard, M. A., Turkheimer, F., Misic, B., Deco, G., Martins, D., & Dipasquale, O. (2023). From neurotransmitters to networks: Transcending organisational hierarchies with molecular-informed functional imaging. *Neuroscience & Biobehavioral Reviews*, *150*, 105193. https://doi.org/10.1016/j.neubiorev.2023.105193

Liu, C., Zhuang, K., Zeitlen, D. C., Chen, Q., Wang, X., Feng, Q., … Qiu, J. (2024). Neural, genetic, and cognitive signatures of creativity. *Communications Biology*, *7*(1), 1324. https://doi.org/10.1038/s42003-024-07007-6

Luppi, A. I., Golkowski, D., Ranft, A., Ilg, R., Jordan, D., Bzdok, D., … Misic, B. (2023, November 13). General anaesthesia reduces the uniqueness of brain connectivity across individuals and across species. https://doi.org/10.1101/2023.11.08.566332

Luppi, A. I., Hansen, J. Y., Adapa, R., Carhart-Harris, R. L., Roseman, L., Timmermann, C., … Stamatakis, E. A. (2023). In vivo mapping of pharmacologically induced functional reorganization onto the human brain’s neurotransmitter landscape. *Science Advances*, *9*(24). https://doi.org/10.1126/sciadv.adf8332

Luppi, A. I., Singleton, S. P., Hansen, J. Y., Jamison, K. W., Bzdok, D., Kuceyeski, A., … Misic, B. (2024). Contributions of network structure, chemoarchitecture and diagnostic categories to transitions between cognitive topographies. *Nature Biomedical Engineering*, *8*(9), 1142–1161. https://doi.org/10.1038/s41551-024-01242-2

Martins, D., Veronese, M., Turkheimer, F. E., Howard, M. A., Williams, S. C. R., & Dipasquale, O. (2022). A candidate neuroimaging biomarker for detection of neurotransmission-related functional alterations and prediction of pharmacological analgesic response in chronic pain. *Brain Communications*, *4*(1). https://doi.org/10.1093/braincomms/fcab302

Morys, F., Tremblay, C., Rahayel, S., Hansen, J. Y., Dai, A., Misic, B., & Dagher, A. (2024). Neural correlates of obesity across the lifespan. *Communications Biology*, *7*(1), 656. https://doi.org/10.1038/s42003-024-06361-9

Pedersen, R., Johansson, J., Nordin, K., Rieckmann, A., Wåhlin, A., Nyberg, L., … Salami, A. (2024). Dopamine D1-Receptor Organization Contributes to Functional Brain Architecture. *The Journal of Neuroscience*, *44*(11), e0621232024. https://doi.org/10.1523/JNEUROSCI.0621-23.2024

Preller, K. H., Burt, J. B., Ji, J. L., Schleifer, C. H., Adkinson, B. D., Stämpfli, P., … Anticevic, A. (2018). Changes in global and thalamic brain connectivity in LSD-induced altered states of consciousness are attributable to the 5-HT2A receptor. *ELife*, *7*. https://doi.org/10.7554/eLife.35082

Saberi, A., Ebneabbasi, A., Rahimi, S., Sarebannejad, S., Sen, Z. D., Graf, H., … Tahmasian, M. (2025). Convergent functional effects of antidepressants in major depressive disorder: a neuroimaging meta-analysis. *Molecular Psychiatry*, *30*(2), 736–751. https://doi.org/10.1038/s41380-024-02780-6

Selvaggi, P., Hawkins, P. C. T., Dipasquale, O., Rizzo, G., Bertolino, A., Dukart, J., … Mehta, M. A. (2019). Increased cerebral blood flow after single dose of antipsychotics in healthy volunteers depends on dopamine D2 receptor density profiles. *NeuroImage*, *188*, 774–784. https://doi.org/10.1016/j.neuroimage.2018.12.028

Shafiei, G., Fulcher, B. D., Voytek, B., Satterthwaite, T. D., Baillet, S., & Misic, B. (2023). Neurophysiological signatures of cortical micro-architecture. *Nature Communications*, *14*(1), 6000. https://doi.org/10.1038/s41467-023-41689-6

Siegel, J. S., Subramanian, S., Perry, D., Kay, B. P., Gordon, E. M., Laumann, T. O., … Dosenbach, N. U. F. (2024). Psilocybin desynchronizes the human brain. *Nature*, *632*(8023), 131–138. https://doi.org/10.1038/s41586-024-07624-5

Singleton, S. P., Luppi, A. I., Carhart-Harris, R. L., Cruzat, J., Roseman, L., Nutt, D. J., … Kuceyeski, A. (2022). Receptor-informed network control theory links LSD and psilocybin to a flattening of the brain’s control energy landscape. *Nature Communications*, *13*(1), 5812. https://doi.org/10.1038/s41467-022-33578-1

Stoliker, D., Novelli, L., Khajehnejad, M., Biabani, M., Barta, T., Greaves, M. D., … Razi, A. (2025, March 11). Psychedelics Align Brain Activity with Context. https://doi.org/10.1101/2025.03.09.642197

Timmermann, C., Roseman, L., Haridas, S., Rosas, F. E., Luan, L., Kettner, H., … Carhart-Harris, R. L. (2023). Human brain effects of DMT assessed via EEG-fMRI. *Proceedings of the National Academy of Sciences*, *120*(13). https://doi.org/10.1073/pnas.2218949120

Vamvakas, A., Lawn, T., Veronese, M., Williams, S. C. R., Tsougos, I., & Howard, M. A. (2022). Neurotransmitter receptor densities are associated with changes in regional Cerebral blood flow during clinical ongoing pain. *Human Brain Mapping*, *43*(17), 5235–5249. https://doi.org/10.1002/hbm.25999

Vignando, M., Ffytche, D., Lewis, S. J. G., Lee, P. H., Chung, S. J., Weil, R. S., … Mehta, M. A. (2022). Mapping brain structural differences and neuroreceptor correlates in Parkinson’s disease visual hallucinations. *Nature Communications*, *13*(1), 519. https://doi.org/10.1038/s41467-022-28087-0

Vohryzek, J., Cabral, J., Lord, L.-D., Fernandes, H. M., Roseman, L., Nutt, D. J., … Kringelbach, M. L. (2024). Brain dynamics predictive of response to psilocybin for treatment-resistant depression. *Brain Communications*, *6*(2). https://doi.org/10.1093/braincomms/fcae049

Xu, X., Zhao, H., Song, Y., Cai, H., Zhao, W., Tang, J., … Yu, Y. (2024). Molecular mechanisms underlying the neural correlates of working memory. *BMC Biology*, *22*(1), 238. https://doi.org/10.1186/s12915-024-02039-0
